# Complete genome sequence of *Desulfomicrobium baculatum* type strain (X^T^)

**DOI:** 10.4056/sigs.13134

**Published:** 2009-07-20

**Authors:** Alex Copeland, Stefan Spring, Markus Göker, Susanne Schneider, Alla Lapidus, Tijana Glavina Del Rio, Hope Tice, Jan-Fang Cheng, Feng Chen, Matt Nolan, David Bruce, Lynne Goodwin, Sam Pitluck, Natalia Ivanova, Konstantinos Mavrommatis, Galina Ovchinnikova, Amrita Pati, Amy Chen, Krishna Palaniappan, Miriam Land, Loren Hauser, Yun-Juan Chang, Cynthia C. Jeffries, Linda Meincke, David Sims, Thomas Brettin, John C. Detter, Cliff Han, Patrick Chain, Jim Bristow, Jonathan A. Eisen, Victor Markowitz, Philip Hugenholtz, Nikos C. Kyrpides, Hans-Peter Klenk, Susan Lucas

**Affiliations:** 1 DOE Joint Genome Institute, Walnut Creek, California, USA; 2 DSMZ - German Collection of Microorganisms and Cell Cultures GmbH, Braunschweig, Germany; 3 Los Alamos National Laboratory, Bioscience Division, Los Alamos, New Mexico USA; 4 Biological Data Management and Technology Center, Lawrence Berkeley National Laboratory, Berkeley, California, USA; 5 Oak Ridge National Laboratory, Oak Ridge, Tennessee, USA; 6 Lawrence Livermore National Laboratory, Livermore, California, USA; 7 University of California Davis Genome Center, Davis, California, USA

**Keywords:** Sulfate reducer, Gram-negative, free-living, non-pathogenic, freshwater, anaerobe, mesophile, *Desulfomicrobiaceae*

## Abstract

*Desulfomicrobium baculatum* is the type species of the genus *Desulfomicrobium*, which is the type genus of the family *Desulfomicrobiaceae*. It is of phylogenetic interest because of the isolated location of the family *Desulfomicrobiaceae* within the order *Desulfovibrionales*. *D. baculatum* strain X^T^ is a Gram-negative, motile, sulfate-reducing bacterium isolated from water-saturated manganese carbonate ore. It is strictly anaerobic and does not require NaCl for growth, although NaCl concentrations up to 6% (w/v) are tolerated. The metabolism is respiratory or fermentative. In the presence of sulfate, pyruvate and lactate are incompletely oxidized to acetate and CO_2_. Here we describe the features of this organism, together with the complete genome sequence and annotation. This is the first completed genome sequence of a member of the deltaproteobacterial family *Desulfomicrobiaceae*, and this 3,942,657 bp long single replicon genome with its 3494 protein-coding and 72 RNA genes is part of the *** G****enomic* *** E****ncyclopedia of* *** B****acteria and* *** A****rchaea * project.

## Introduction

**Figure 1:**
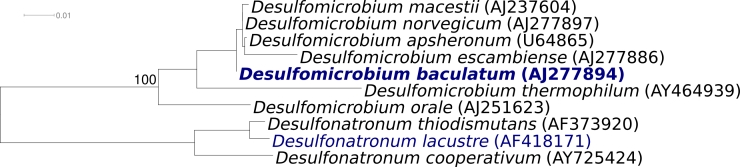
Phylogenetic tree of *D. baculatum* strain X^T^ and all type strains of species within members of the family *Desulfomicrobiaceae*, inferred from 1,457 aligned characters [4,5] of the 16S rRNA gene sequence under the maximum likelihood criterion [6]. The tree was rooted with all members from the *Desulfonatronaceae*, another family in the order *Desulfovibrionales*. The branches are scaled in terms of the expected number of substitutions per site. Numbers above branches are support values from 1,000 bootstrap replicates if larger than 60%. Strains with a genome-sequencing project registered in GOLD [7] are printed in blue; published genomes in bold.

Strain X^T^ (DSM 4028 = CCUG 34229 = VKM B-1378) is the type strain of the species *Desulfomicrobium baculatum*, which is the type species of the genus *Desulfomicrobium*. Strain X^T^ was first described as *Desulfovibrio baculatus* by Rozanova and Nazina [1,2], and later transferred to the novel genus *Desulfomicrobium* (currently containing seven species) [3] (Figure 1) because several phenotypic traits were not consistent with the definition of the genus *Desulfovibrio*. In 1998 the species epithet was corrected to *D. baculatum* [8]. Three accompanying strains have been described in addition to strain X^T^: Strain H.L21 (DSM 2555) was isolated from anoxic intertidal sediment at the Ems-Dollard Estuary, Netherlands (16S rRNA gene accession AJ277895) [9], strain 5174 (DSM 17142) was isolated from a forest pond near Braunschweig, Germany (16S rRNA gene accession AJ277896) [10], and strain 9974 (DSM 17143) was isolated as a contaminating chemotrophic bacterium from a culture of a green sulfur bacterium designated *’Chloropseudomonas ethylica*’ N2 [10]. These strains were tentatively affiliated with the species *D. baculatum* based on some phenotypic traits. Although 16S rRNA gene sequence data are now available for two strains, a definitive affiliation of strains to the species *Desulfomicrobium* requires supplementary DNA-DNA hybridization experiments due to the observed high similarity values of 16S rRNA gene sequences among distinct species of this genus [11]. Other isolates and clones related to the species were isolated from production waters of a low-temperature biodegraded oil reservoir in Canada [12], and wastewater from penicillin G production in China (clone B19 EU234202). Screening of environmental genomic samples and surveys reported at the NCBI BLAST server indicated no closely related phylotypes that can be linked to the species. Here we present a summary classification and a set of features for *D. baculatum* strain X^T^ (Table 1), together with the description of the complete genomic sequencing and annotation.

**Table 1:** Classification and general features of *D. baculatum* X^T^ in accordance to the MIGS recommendations [13]

MIGS ID	Property	Term	Evidence code
	Current classification	Domain *Bacteria* Phylum *Proteoobacteria* Class *Deltaproteobacteria* Order *Desulfovibrionales* Family *Desulfomicrobiaceae* Genus *Desulfomicrobium* Species *Desulfomicrobium baculatum* Type strain X	TAS [14] TAS [14] TAS [14] TAS [1] TAS [1] TAS [1]
	Gram stain	negative	TAS [1]
	Cell shape	rod-shaped	TAS [1]
	Motility	motile, single polar flagellum	TAS [1]
	Sporulation	non-sporulating	TAS [1]
	Temperature range	mesophilic	TAS [1]
	Optimum temperature	28-37°C	TAS [1]
	Salinity	10 g NaCl/l	TAS [1]
MIGS-22	Oxygen requirement	strictly anerobic	TAS [1]
	Carbon source	lactate, pyruvate, malate, fumarate	TAS [1,12]
	Energy source	formate, H_2_	TAS [1]
MIGS-6	Habitat	freshwater to brackish anoxic sediments	TAS [1]
MIGS-15	Biotic relationship	free-living	NAS
MIGS-14	Pathogenicity	none	NAS
	Biosafety level	1	TAS [15]
	Isolation	water-saturated manganese carbonate ore	TAS [1]
MIGS-4	Geographic location	not reported	
MIGS-5	Sample collection time	1975 or earlier	IDA
MIGS-4.1 MIGS-4.2	Latitude – Longitude	not reported	
MIGS-4.3	Depth	not reported	
MIGS-4.4	Altitude	not reported	

Evidence codes – IDA: Inferred from Direct Assay (first time in publication); TAS: Traceable Author Statement (i.e., a direct report exists in the literature); NAS: Non-traceable Author Statement (i.e., not directly observed for the living, isolated sample, but based on a generally accepted property for the species, or anecdotal evidence). These evidence codes are from the Gene Ontology project [16]. If the evidence code is IDA, then the property was directly observed for a live isolate by one of the authors or an expert mentioned in the acknowledgements.

## Classification and features

**Figure 2:**
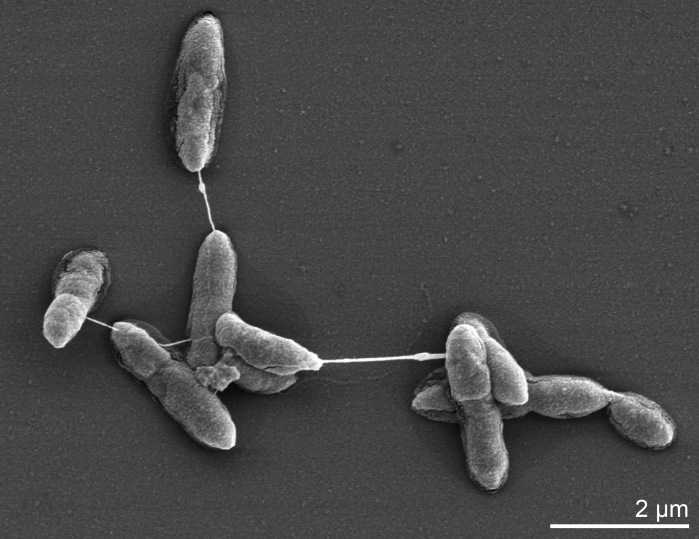
Scanning electron micrograph of *D. baculatum* X^T^ (Manfred Rohde, Helmholtz Centre for Infection Biology, Braunschweig)

Cells of *D. baculatum* strain X^T^ are short rods with rounded ends of 0.6 x 1-2 µm (Figure 2). Cells stain Gram-negative, are motile by a single polar flagellum, and do not form endospores. The metabolism is strictly anaerobic and can be respiratory or fermentative [3,17]. Temperature range for growth is 2-41°C (optimum 28-37°C) and NaCl concentrations of 0-6% (w/v) are tolerated (optimum 1% w/v). Sulfate, sulfite and thiosulfate are used as electron acceptors and are reduced to H_2_S. Nitrate is not reduced. Simple organic compounds are incompletely oxidized to acetate [3]. Malate, fumarate and pyruvate can be fermented with succinate and acetate as end products. Carbohydrates are not fermented. Vitamins are not required for growth [3]. *D. baculatum* strain 9974 (DSM 1743) is also able to use ethanol as a substrate [18] and sulfur as an electron acceptor [10]. The use of ethanol as an electron donor for sulfate respiration depends on supplementing the medium with the trace elements tungstate or molybdate [18]. Sulfate uptake in symport with sodium ions has been shown in strain 9974, unlike in other fresh water sulfate reducers which use protons [19]. Distinctive features of *D. baculatum* strain X^T^ are: (i) NaCl is not required for growth [3], (ii) fermentation of fumarate and malate to succinate and acetate is preferred against utilization of these substrates as electron donors for sulfate reduction [17], (iii) sulfur is not used as an electron acceptor and (IV) molecular nitrogen can be assimilated [3].

A desulfoviridin-type dissimilatory sulfite reductase, which is a hallmark feature of the genus Desulfovibrio, is absent in strain X^T^, however a sulfite reductase of the desulforubidin-type was reported for strain 9974 [20]. Cells of D. baculatum strain X^T^ contain c- and b-type cytochromes [3]. The tetraheme cytochrome c_3_ of strain 9974, which is thought to play a role in sulfur reduction and the coupling of electron transfer to hydrogenases, has been analyzed in some detail using advanced biophysical methods [21-23]. Strain 9974 also contains several distinct [NiFeSe] hydrogenases that are located in different cellular compartments [24]. The crystal structure of the periplasmic [NiFeSe] hydrogenase of this strain has been determined [25] and it is proposed that the selenium ion in the active center plays a role in the oxygen-tolerant hydrogen production of this enzyme, which distinguishes it from most [NiFe] hydrogenases [26]. An active selenocysteine system for usage of the 21^st^ amino acid has been studied in detail for D. baculatum strain 9974 [27-29]. Pyridoxal-5’-phosphate, the prosthetic group of selenocysteine synthases, is bound to a distinct lysine residue (Lys295) within the active site of the enzyme of this strain [28].

Figure 1 shows the phylogenetic neighborhood of *D. baculatum* strain X^T^ in a 16S rRNA based tree. Analysis of the two 16S rRNA gene sequences in the genome of strain X^T^ indicated that the two genes are almost identical (1 bp difference), and that both genes differed by one nucleotide from the previously published 16S rRNA sequence generated from DSM 4028 (AJ277894).

## Chemotaxonomy

The cellular fatty acid patterns of *D. baculatum* strain X^T^ and the accompanying strains 5174, 9974 and H.L21 [30] were found to be dominated by anteiso- (ai) and iso-methyl branched unsaturated and saturated fatty acids. The most abundant fatty acid is iso-17:1 cis7 (24.2-28.6%), followed by 18:1 cis11 (6.4-12.2%), iso-15:0 (8.2-11.6%), ai-17:0 (4.5-8.3%), ai-15:0 (5.2-7.7%), 18:0 (3.9-7.1%) and 16:0 (3.6-5.7%). Less abundant fatty acids are iso-15:1 (3.1–4.0%), 16:1 cis7 (2.2–5.0%), ai-17:1 (2.4–4.1%), 18:1 cis9 (2.6–4.3%), iso-16:1 (0.5–2.2%), and 17:0 (0.2-0.3%). Branched chain, hydroxylated fatty acids are also present, 3-OH iso-15:0 (1.4–2.4%), 3-OH ai-15:0 (0.7–1.2%), and 3-OH iso-17:0 (1.2–2.2%), which may be derived from a lipopolysaccharide. The polar lipid composition of *D. baculatum* strain X^T^ has not been investigated. The respiratory quinone composition of *D. baculatum* strain X^T^ has also not been investigated, but the presence of MK-6 has been reported in *D. macestii* and *D. norvegicum* [11,31].

## Genome sequencing and annotation

### Genome project history

This organism was selected for sequencing on the basis of its phylogenetic position, and is part of the Genomic Encyclopedia of Bacteria and Archaea project. The genome project is deposited in the Genomes OnLine Database [7] and the complete genome sequence (CP001629) is deposited in GenBank. Sequencing, finishing and annotation were performed by the DOE Joint Genome Institute (JGI). A summary of the project information is shown in Table 2.

**Table 2:** Genome sequencing project information

MIGS ID	Property	Term
MIGS-31	Finishing quality	Finished
MIGS-28	Libraries used	Two genomic libraries: one 8 kb Sanger pMCL200 library and one 454 pyrosequence standard library
MIGS-29	Sequencing platforms	ABI3730, 454 GS FLX
MIGS-31.2	Sequencing coverage	6.8x Sanger; 30.4x pyrosequence
MIGS-20	Assemblers	Newbler version 1.1.02.15, phrap
MIGS-32	Gene calling method	Prodigal
	INSDC / Genbank ID	CP001629
	Genbank Date of Release	not yet
	GOLD ID	Gc01026
	NCBI project ID	29527
	Database: IMG-GEBA	2501416908
MIGS -13	Source material identifier	DSM 4028
	Project relevance	Tree of Life, GEBA

### Growth conditions and DNA isolation

D. baculatum strain X^T^ (DSM 4028) was grown in DSMZ medium 63 at 30°C. DNA was isolated from 1-1.5 g of cell paste using Qiagen Genomic 500 DNA Kit (Qiagen, Hilden, Germany) with a modified protocol for cell lysis, adding 100 µl lysozyme; 500 µl achromopeptidase, lysostaphin, mutanolysin, each, to standard lysis solution, but reducing proteinase K to 160µl, only. Incubation over night at 35°C.

### Genome sequencing and assembly

The genome was sequenced using a combination of Sanger and 454 sequencing platforms. All general aspects of library construction and sequencing performed at the JGI can be found at the JGI website. 454 Pyrosequencing reads were assembled using the Newbler assembler version 1.1.02.15 (Roche). Large Newbler contigs were broken into 4,375 overlapping fragments of 1,000 bp and entered into assembly as pseudo-reads. The sequences were assigned quality scores based on Newbler consensus q-scores with modifications to account for overlap redundancy and to adjust inflated q-scores. A hybrid 454/Sanger assembly was made using the parallel phrap assembler (High Performance Software, LLC). Possible mis-assemblies were corrected with Dupfinisher or transposon bombing of bridging clones [32]. Gaps between contigs were closed by editing in Consed, custom primer walk or PCR amplification. 731 Sanger finishing reads were produced to close gaps, to resolve repetitive regions, and to raise the quality of the finished sequence. The error rate of the completed genome sequence is less than 1 in 100,000. Together all sequence types provided 37.2 x coverage of the genome.

### Genome annotation

Genes were identified using Prodigal [33] as part of the Oak Ridge National Laboratory genome annotation pipeline, followed by a round of manual curation using JGI’s GenePRIMP pipeline [34]. The predicted CDSs were translated and used to search the National Center for Biotechnology Information (NCBI) nonredundant database, UniProt, TIGRFam, Pfam, PRIAM, KEGG, COG, and InterPro databases. Additional gene prediction analysis and functional annotation were performed within the Integrated Microbial Genomes (IMG-ER) platform.

### Genome properties

**Figure 3:**
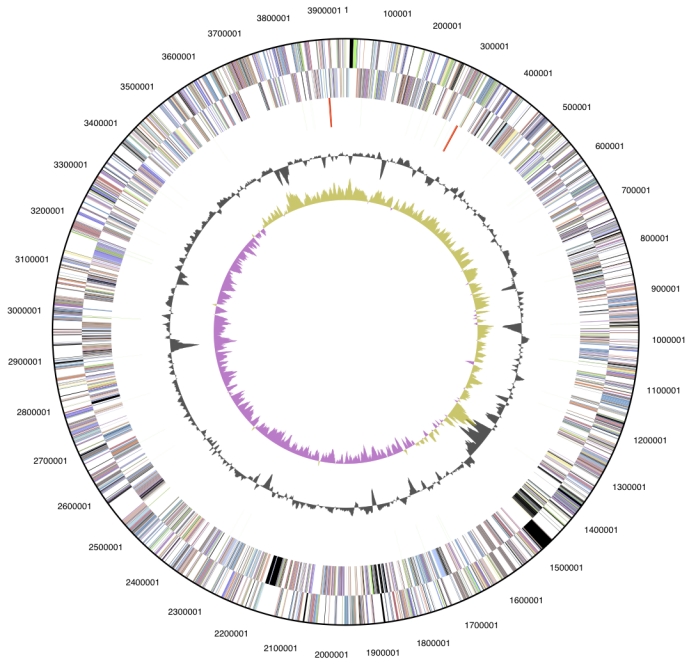
**Graphical circular map of the genome.** From outside to the center: Genes on forward strand (color by COG categories), Genes on reverse strand (color by COG categories), RNA genes (tRNAs green, rRNAs red, other RNAs black), GC content, GC skew.

The genome is 3,942,657 bp long and comprises one circular chromosome with a 58.7% GC content (Table 3 and Figure 3). Of the 3,565 genes predicted, 3,494 were protein coding genes, and 71 RNAs; 58 pseudogenes were also identified. 74.9% of the genes were assigned a putative function while the remaining ones were annotated as hypothetical proteins. The properties and the statistics of the genome are summarized in Table 3. The distribution of genes into COGs functional categories is presented in Table 4.

**Table 3:** Genome Statistics

Attribute	Value	% of Total
Genome size (bp)	3,942,657	
DNA Coding region (bp)	3,572,336	90.61%
DNA G+C content (bp)	2,312,250	58.65%
Number of replicons	1	
Extrachromosomal elements	0	
Total genes	3565	
RNA genes	71	2.02%
rRNA operons	2	
Protein-coding genes	3494	97.98%
Pseudo genes	58	1.63%
Genes with function prediction	2675	75.01%
Genes in paralog clusters	357	12.82%
Genes assigned to COGs	2689	75.41%
Genes assigned Pfam domains	2688	75.38%
Genes with signal peptides	723	20.27%
Genes with transmembrane helices	897	25.15%
CRISPR repeats	0	

**Table 4:** Number of genes associated with the 21 general COG functional categories

Code	Value	%	Description
J	166	4.8	Translation, ribosomal structure and biogenesis
A	0	0.0	RNA processing and modification
K	154	4.4	Transcription
L	116	3.3	Replication, recombination and repair
B	3	0.1	Chromatin structure and dynamics
D	35	1.0	Cell cycle control, mitosis and meiosis
Y	0	0.0	Nuclear structure
V	38	1.1	Defense mechanisms
T	325	9.3	Signal transduction mechanisms
M	221	6.3	Cell wall/membrane biogenesis
N	108	3.1	Cell motility
Z	0	0.0	Cytoskeleton

**Table 4:** Number of genes associated with the 21 general COG functional categories (cont.)

Code	Value	%	Description
W	0	0.0	Extracellular structures
U	82	2.3	Intracellular trafficking and secretion
O	122	3.5	Posttranslational modification, protein turnover, chaperones
C	241	6.9	Energy production and conversion
G	126	3.6	Carbohydrate transport and metabolism
E	266	7.6	Amino acid transport and metabolism
F	68	1.9	Nucleotide transport and metabolism
H	135	3.9	Coenzyme transport and metabolism
I	52	1.5	Lipid transport and metabolism
P	137	3.9	Inorganic ion transport and metabolism
Q	34	1.0	Secondary metabolites biosynthesis, transport and catabolism
R	319	9.1	General function prediction only
S	221	6.3	Function unknown
-	805	23.0	Not in COGs
